# Hierarchical Micro–Mesoporous ZnO–SiO_2_/Carbon Composites: Synthesis, Structural Characterisation, and High-Capacity Adsorption of Cationic Organic Pollutants from Water

**DOI:** 10.3390/molecules31122079

**Published:** 2026-06-13

**Authors:** Mariia Galaburda, Małgorzata Wasilewska, Elżbieta Grządka, Jolanta Kutkowska

**Affiliations:** 1 Department of Physical Chemistry, Institute of Chemical Sciences, Faculty of Chemistry, Maria Curie-Skłodowska University, M. Curie-Skłodowska Sq 3, 20-031 Lublin, Poland; mariia.galaburda@gmail.com (M.G.); malgorzata.wasilewska@mail.umcs.pl (M.W.); 2 Chuiko Institute of Surface Chemistry, NAS of Ukraine, 17 General Naumov Str., 03164 Kyiv, Ukraine; 3 Department of Genetics and Microbiology, Maria Curie-Skłodowska University, Akademicka 19, 20-031 Lublin, Poland; jolanta.kutkowska@mail.umcs.pl

**Keywords:** carbon sorbents, adsorption, non-leaching character, zinc ion retention, biofouling resistance potential, zinc oxide nanoparticles

## Abstract

Hierarchical ZnO–SiO_2_/carbon composites (C-Zn1, C-Zn2, C-Zn3) were synthesised via the carbonisation of resorcinol–formaldehyde gels in the presence of ZnO-modified fumed silica, and characterised by N_2_ adsorption–desorption, FTIR, XRD, SEM, and zeta potential analysis. The composites exhibited hierarchical micro–mesoporous structures with BET surface areas of 467–499 m^2^ g^−1^; the non-microporous volume fraction increased from 0.09 (reference carbon RFC, 545 m^2^ g^−1^) to 0.54–0.63 upon ZnO–SiO_2_ incorporation. Adsorption of methylene blue (MB), crystal violet (CV), and rhodamine 6G (R6G) followed the Marczewski–Jaroniec isotherm model. Maximum adsorption capacities for the best-performing composite (C-Zn1) reached 1.22 mmol g^−1^ for MB, 1.04 mmol g^−1^ for CV, and 0.63 mmol g^−1^ for R6G, compared to 1.32, 1.17, and 0.67 mmol g^−1^ for unmodified RFC. Kinetic analysis revealed up to 3.5-fold faster adsorption rates for C-Zn1 relative to RFC (for CV and R6G), attributed to enhanced diffusion through mesoporous channels while preserving the micropore-driven capacity. Agar well-diffusion assays against four bacterial strains showed no inhibition zones for any composite, indicating that no biologically active concentration of zinc species was released under the assay conditions. The proposed approach yields composites with enhanced adsorption kinetics, preserved capacity, and confirmed non-leaching character, positioning them as effective candidates for water purification.

## 1. Introduction

The contamination of aquatic environments with persistent organic micropollutants represents one of the most pressing challenges in environmental chemistry and public health. Industrial effluents, agricultural runoff, and inadequately treated municipal wastewater continuously release recalcitrant cationic species, synthetic dyes, pharmaceuticals, endocrine disruptors, and pesticides, into surface and groundwater systems [1,2]. Their persistence, bioaccumulation, and toxicity at trace concentrations render them poorly addressed by conventional coagulation, sedimentation, and biological treatment, driving sustained demand for advanced adsorbents.

Carbon-based sorbents, particularly resorcinol–formaldehyde-derived carbons (RFC), occupy a central role in water treatment due to their exceptional surface area, tuneable surface functionality, and strong affinity for aromatic species via π–π stacking, electrostatic attraction, and pore-filling mechanisms [3,4,5]. Hierarchical architectures combining micropores and mesopores (2–50 nm) [6] markedly improve the diffusion kinetics and adsorption capacity for medium- to large-sized contaminants relative to purely microporous frameworks [1]. RFCs are particularly attractive precursors because their sol–gel polycondensation chemistry enables direct control over pore architecture via solvent composition, pH, and the inclusion of sacrificial inorganic additives.

A critical vulnerability of carbonaceous sorbents in long-term operation is progressive microbial biofouling: bacteria rapidly colonise the high-surface-area matrix, forming biofilms that block pores, reduce accessible surface area, increase hydraulic resistance in packed beds, and shorten sorbent lifetime [7,8]. More critically, a biofilm-laden carbon filter can release pathogenic bacteria into downstream water, converting the purification element into a contamination vector independent of influent quality and not addressed by upstream disinfection [7].

Zinc oxide nanoparticles have attracted significant interest as broad-spectrum antimicrobials active against Gram-positive bacteria (*Staphylococcus aureus*, *Enterococcus faecalis*), Gram-negative species (*Escherichia coli*, *Enterobacter cloacae*), and fungal pathogens [7,9,10]. Their multi-mechanistic biocidal action, surface-catalysed generation of reactive oxygen species (ROS), direct membrane disruption, and localised Zn^2+^ release, hinders the development of microbial resistance [9,11]. ZnO is also classified as generally recognised as safe (GRAS) by the U.S. FDA (21 CFR §182.8991) [12], and approved for food-contact and water-treatment applications, placing it in a uniquely favourable position relative to CuO or silver nanoparticles. Beyond its biocidal role, ZnO exhibits photocatalytic activity under UV and near-visible light, enabling oxidative mineralisation of the adsorbed contaminants and pathways toward sorbent self-cleaning [13]. Its integration with carbonaceous substrates is therefore an established strategy for hybrid materials combining adsorption, antifouling, and photocatalytic functionalities [8,14,15,16]. The principal limitation remains uncontrolled Zn^2+^ leaching from loosely bound or aggregated ZnO.

The use of ZnO-functionalised fumed silica (ZnO–SiO_2_) as a bifunctional modifier of RFC offers a coherent solution [17,18]. Fumed silica (S_BET_ ≈ 378 m^2^ g^−1^ for ORISIL-380) provides a high-surface-area support on which zinc species are anchored via condensation with silanol (Si–OH) groups, forming thermally and hydrolytically stable Si–O–Zn linkages [18,19]. This chemical immobilisation, fundamentally distinct from the weak physisorption typical of carbon–ZnO composites, is expected to suppress ion leaching across a broad pH range. A low ZnO loading of 0.2 mmol Zn g^−1^ SiO_2_ was selected to favour ultrafine, X-ray amorphous ZnO with maximal surface dispersion, as crystalline ZnO becomes detectable in X-ray diffraction only at loadings ≥ 3 mmol Zn g^−1^ SiO_2_ [19]. Concurrently, ZnO–SiO_2_ aggregates dispersed in the RF gel act as sacrificial porogens during pyrolysis, generating interconnected mesopore channels that complement the intrinsic microporosity of RFC [17,18].

We hypothesised that incorporating chemically anchored ZnO nanostructures into an RFC matrix via a ZnO–SiO_2_ modifier would simultaneously generate diffusion-accessible mesopore channels for the efficient uptake of bulky organic pollutants and create a biostatic surface suppressing bacterial colonisation, without releasing zinc ions into the aqueous phase. This dual functionality was expected to arise from strong Si–O–Zn interfacial bonding, decoupling surface antimicrobial character from metal ion leaching [19,20]. Accordingly, this work aims to synthesise a series of ZnO–SiO_2_-modified carbon composites at varied inorganic loadings, characterise their structural, textural, and electrokinetic properties, evaluate their adsorption performance toward cationic organic model pollutants of differing size and hydrophilicity, and assess their non-leaching character under conditions relevant to water purification.

## 2. Results and Discussion

### 2.1. Structural and Spectroscopic Characterisation (FTIR and XRD)

**Figure 1:**
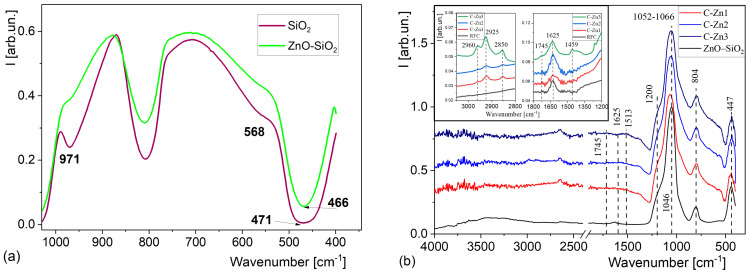
(**a**) FTIR spectra of pristine SiO_2_ and the ZnO–SiO_2_ modifier in the 400–1050 cm^−1^ region. (**b**) ATR FTIR spectra of the ZnO–SiO_2_ modifier and the carbon composites C-Zn1, C-Zn2, and C-Zn3 in the 400–4000 cm^−1^ range. Insets show KBr-pellet transmission spectra of RFC, C-Zn1, C-Zn2, and C-Zn3 in the 2800–3050 cm^−1^ and 1200–1800 cm^−1^ regions.

**Figure 2:**
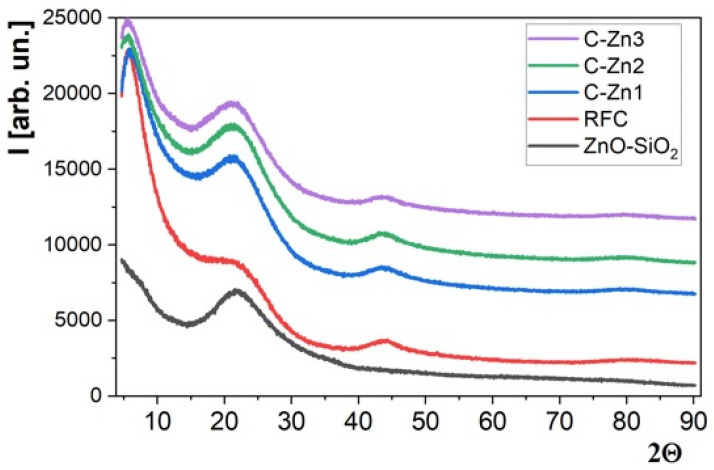
XRD patterns of ZnO–SiO_2_, RFC, C-Zn1, C-Zn2, and C-Zn3 in the 5–90° 2θ range (curves vertically offset for clarity).

The structural and surface chemical evolution from pristine fumed SiO_2_ through the ZnO–SiO_2_ modifier to the final carbon composites was probed by complementary Fourier transform infrared (FTIR) spectroscopy in attenuated total reflectance (ATR) and KBr-pellet modes (Figure 1) and by X-ray diffraction (XRD, Figure 2).

In the 400–1050 cm^−1^ region (Figure 1a), liquid-phase modification of fumed SiO_2_ with zinc acetate and calcination at 600 °C produced three diagnostic changes. The band at 971 cm^−1^, assigned to isolated silanol (Si–OH) stretching, decreased markedly upon Zn incorporation, evidencing condensation of surface hydroxyl groups with zinc species and the formation of Si–O–Zn linkages [21]. The O–Si–O bending mode shifted from 471 to 466 cm^−1^, consistent with the incorporation of heavier Zn atoms into the silica network, while slight broadening and asymmetry near 568 cm^−1^ reflected structural distortion and the formation of highly dispersed ZnO anchored to the silica surface. The low Zn loading (0.2 mmol Zn g^−1^ SiO_2_) suppressed bulk crystallisation in favour of ultrafine ZnO domains [20].

After pyrolysis of the resorcinol–formaldehyde (RF) gel embedded with ZnO–SiO_2_ at 800 °C, the ATR FTIR spectra of C-Zn1, C-Zn2, and C-Zn3 (Figure 1b) retained the three diagnostic Si–O–Si vibrations of the inorganic framework: asymmetric stretching (*ν_as_*) at 1052–1066 cm^−1^, symmetric stretching (*ν_s_*) at 804 cm^−1^, and bending (*δ*) at 447 cm^−1^. Relative to the precursor ZnO–SiO_2_ (*ν_as_* at 1046 cm^−1^), the systematic shift of *ν_as_* toward higher wavenumbers reflects enhanced condensation of the silica network during carbonisation, suggesting the preservation of a strong interfacial interaction between the Zn-containing species and the silica framework after high-temperature treatment [22,23].

The carbon phase contributed a broad *ν*(O–H) absorption near 3400 cm^−1^ (hydrogen-bonded silanol, carboxylic, and phenolic groups), a phenolic ν(C–O) band near ~1200 cm^−1^, and a weak ν(C=O) at ~1745 cm^−1^ [24]. Furthermore, the bands at ~1513 and ~1625 cm^−1^ can be attributed to aromatic skeletal vibrations mixed with the stretching of highly conjugated C=O groups (such as quinones) within the carbon matrix. The weak ν(C=O) band was absent from the ZnO–SiO_2_ spectrum, confirming its origin from residual –COOH and lactone groups at carbon edge sites formed by incomplete carbonisation of the hydroxyl-rich RF network. Because intense Si–O–Si absorption obscures these weaker carbon bands in ATR mode, KBr-pellet transmission spectra were additionally recorded with sample-to-KBr ratios optimised for each composite (insets in Figure 1b). The 1200–1800 cm^−1^ inset resolves the ν(C=O) of the carboxylic/lactone groups (~1745 cm^−1^), aromatic and conjugated skeletal vibrations (~1625 cm^−1^), and *δ*(CH_2_) bending (~1459 cm^−1^). The 2800–3050 cm^−1^ inset displays the asymmetric and symmetric C–H stretching of residual aliphatic –CH_2_– and –CH_3_ fragments at ~2960, ~2925, and ~2850 cm^−1^. Together, these signatures confirm that the pyrolysed material retains acidic oxygen functionalities (–COOH, lactones, phenolic –OH) and residual sp^3^ aliphatic fragments, which govern the acidic surface character and the predominantly negative zeta potential of the composites (Section 2.4).

The XRD patterns of all samples (Figure 2) exhibited diffuse halos at ~20–22° and ~43° 2θ, characteristic of amorphous SiO_2_ and turbostratic carbon, with no reflections of crystalline ZnO. This indicates a high dispersion of Zn species or their incorporation into the amorphous network at the low loadings employed [19,25]. Furthermore, the absence of XRD reflections attributable to metallic zinc (which would appear as sharp peaks at 36.3°, 38.9°, and 43.2° 2θ) suggests that carbonisation at 800 °C under nitrogen did not induce widespread carbothermal reduction of the Zn species. The consistent EDS detection of zinc across all composite surface areas (Section 2.3) further confirms its retention within the matrix. While the carbon matrix provides a reducing environment at high temperatures, the strong Si–O–Zn interfacial linkages (as confirmed by FTIR) likely stabilise the Zn^2+^ centres. Previous high-resolution X-ray photoelectron spectroscopy (XPS) studies on analogous ZnO–SiO_2_ nanocomposites under identical sol–gel synthesis conditions confirmed a stable tetrahedral Zn^2+^ coordination [20,25]. The robust retention of this immobilised Zn^2+^ state in the present C-Zn composites is strongly corroborated by the complete absence of soluble zinc release during the antimicrobial assays (Section 2.6).

### 2.2. Textural Characteristics

**Figure 3:**
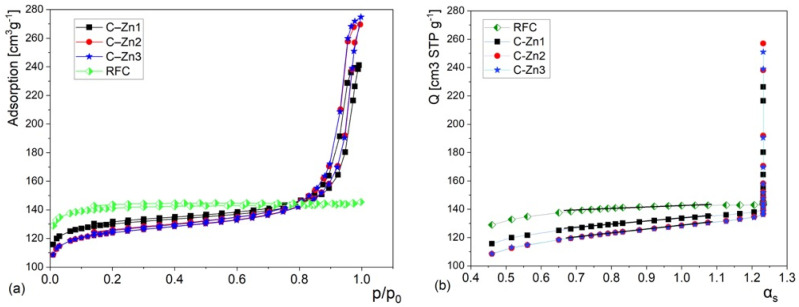
(**a**) N_2_ adsorption–desorption isotherms and (**b**) comparison plots of the adsorbed amount against the reduced adsorption α_s_ of a nonporous-carbon reference [26].

The N_2_ adsorption–desorption isotherms and the corresponding comparison plots are shown in Figure 3. The unmodified RFC reference displayed a Type I(b) isotherm (IUPAC 2015) [6]. A steep uptake at p/p_0_ < 0.05 followed by a near-horizontal plateau extending to p/p_0_ ≈ 0.9 is characteristic of a predominantly microporous solid with pore widths up to 2 nm. No discernible hysteresis was observed, confirming the essentially complete absence of an extended mesopore network.

In contrast, the C-Zn1, C-Zn2 and C-Zn3 composites showed isotherms that combined Type I and Type IV(a) characteristics. Micropore filling at p/p_0_ < 0.05 remained visible, but its relative contribution decreased with increasing ZnO–SiO_2_ loading, as evident directly from the lower low-pressure uptake of C-Zn2 and C-Zn3 compared with C-Zn1 (the adsorbed amount at p/p_0_ = 0.1 was 127 cm^3^ STP g^−1^ for C-Zn1 against 120 cm^3^ STP g^−1^ for C-Zn2 and C-Zn3). After a gently rising multilayer region, all three composites displayed a pronounced uptake at p/p_0_ > 0.85 with a weak hysteresis loop. The fact that the steep rise occurs only at high relative pressure shows that the additional adsorption takes place predominantly in wide mesopores and inter-particle voids rather than in a uniform, narrow mesopore network. The weak high-pressure hysteresis is consistent with H2(b)/H3-type behaviour produced by loosely packed, partially interconnected porosity.

The comparison plots (Figure 3b) were linear over the fitted interval (R^2^ = 0.995–0.999 for the composites, and 0.93 for RFC). The intercept of each fit gives the micropore volume (Γ_micro_) and the slope gives the non-microporous surface area. Two features of the fitted parameters (Table 1) matched the qualitative reading of the isotherms point by point. First, C-Zn1 had the largest micropore volume of the three composites (V_micro_ = 0.172 cm^3^ g^−1^ versus 0.156 cm^3^ g^−1^ for both C-Zn2 and C-Zn3), consistent with its higher low-pressure uptake. Second, C-Zn1 had the smallest non-microporous surface area (S_ext_ = 46 m^2^ g^−1^ versus 57 and 56 m^2^ g^−1^ for C-Zn2 and C-Zn3), consistent with the gentler slope of its comparison plot and of its multilayer branch. RFC, as expected, combined the largest micropore volume (0.204 cm^3^ g^−1^) with a negligible external area (22 m^2^ g^−1^).

The total pore volume increased from 0.225 cm^3^ g^−1^ (RFC) to 0.37–0.43 cm^3^ g^−1^ in the composites, and the non-microporous volume (V_p_ − V_micro_) from 0.02 to 0.20–0.27 cm^3^ g^−1^. The apparent BET surface area decreased moderately, from 545 to 467–499 m^2^ g^−1^, because the loss of high-area microporous surface is only partly compensated by the developing external surface. In terms of pore volume, however, the non-microporous fraction became dominant: (V_p_ − V_micro_)/V_p_ rose from 0.09 (RFC) to 0.54–0.63 (composites), and the average hydraulic diameter D_p_ = 4V_p_/S_BET_ (a volume-weighted mean that underestimates the actual mesopore width) increased from 1.65 to 2.99–3.59 nm. Crucially, the microporous network responsible for the high adsorption capacity was preserved (S_micro_ = 410–452 m^2^ g^−1^ in the composites), while the additional wider porosity provided the diffusion-accessible transport pathways exploited during dye adsorption (Section 2.5).

Finally, the pore-forming role of the ZnO–SiO_2_ phase is supported by the water-matched comparison. Although increasing the suspension volume raised both the inorganic loading and the water content of the reaction mixture, C-Zn1 contained less water (27.1 g) than RFC (~35 g) yet developed markedly higher non-microporous porosity (V_p_ − V_micro_)/V_p_ = 0.54 versus 0.09). This difference cannot be attributed to water content alone and demonstrates that the ZnO–SiO_2_ composite aggregates act as in situ porogens during gel formation and carbonisation.

### 2.3. SEM Analysis

**Figure 4:**
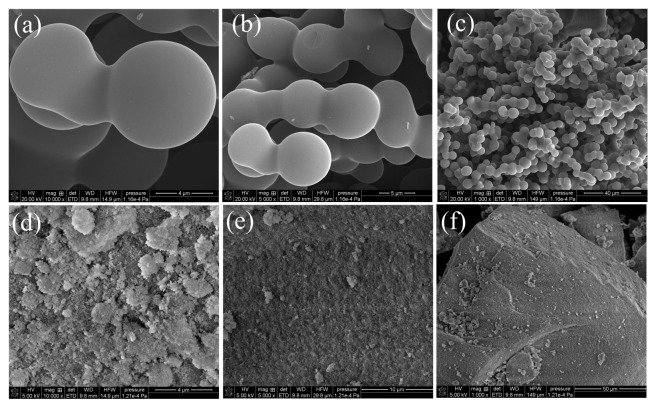
SEM micrographs of the RFC (**a**–**c**) and ZnO–SiO_2_ (**d**–**f**).

Pyrolysis of the RF polymer at 800 °C produced a morphology of fused, globular carbon particles with sphere diameters of 2–5 µm, assembled into grape-like clusters extending to 20 µm or more (Figure 4a–c). The external surface of these spheres was smooth and featureless at the SEM resolution scale, consistent with a relatively dense carbonised outer surface. Crucially, the high specific surface area of RFC (S_BET_ = 545 m^2^ g^−1^) originated predominantly from the internal micropore network (mean hydraulic diameter D_p_ = 1.65 nm, Table 1) and was invisible to SEM, suggesting that the dominant porosity originates from the internal carbon structure rather than from externally visible surface features.

The ZnO–SiO_2_ modifier presented a rough, highly textured surface formed by the aggregation of primary SiO_2_ particles (~7 nm for ORISIL-380) into fractal-like agglomerates of sub-micron to micron dimensions (Figure 4d–f). ZnO, present at only 1.6 wt.% within this material at a loading of 0.2 mmol Zn g^−1^ SiO_2_, was anchored as ultrafine nanostructures below the resolution limit of conventional SEM and below reliable EDS detection at such low concentration. The observed sub-micron roughness therefore corresponds predominantly to SiO_2_ agglomerates rather than to ZnO particles.

**Figure 5:**
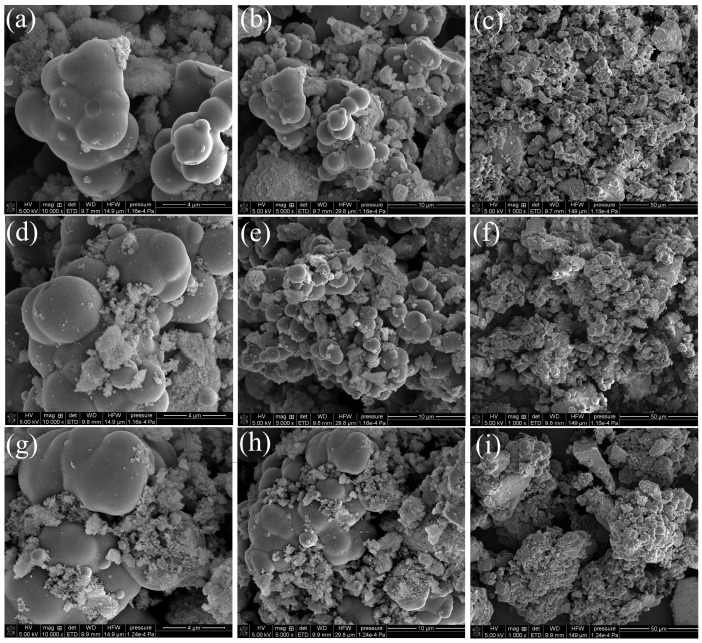
SEM micrographs of the C-Zn1 (**a**–**c**), C-Zn2 (**d**–**f**), and C-Zn3 (**g**–**i**) composites.

In the carbon composites, the sphere surfaces became progressively rougher and more heterogeneous with increasing ZnO–SiO_2_ loading (Figure 5). In C-Zn1 (Figure 5a–c), the carbon spheres remained discernible and were partially coated by isolated SiO_2_-rich agglomerates, containing highly dispersed Zn species. In C-Zn2 (Figure 5d–f), the spheres became almost fully covered by densely distributed SiO_2_-rich agglomerates that obscured much of the underlying carbon morphology. In C-Zn3 (Figure 5g–i), with the highest SiO_2_ loading (26.2 wt.%, Table 2), the carbon framework was largely concealed by a near-continuous inorganic network bridging adjacent spheres. The progressive coverage scales with the SiO_2_ weight fraction, while the internal micropore network responsible for the dominant fraction of S_BET_ is preserved within the carbon bulk.

EDS elemental analysis was conducted on four representative surface areas of each sample. The RFC reference comprised nearly pure carbon (98.1 ± 0.5 wt.%) with trace oxygen, silicon below the detection threshold, and no zinc. In contrast, silicon (4.7–21.1 wt.%) and zinc (0.11–0.20 wt.%) were consistently detected across all areas of the C-Zn1, C-Zn2, and C-Zn3 composites, confirming successful incorporation of the inorganic phase. The large standard deviation of Si content (3.9–6.1 wt.%) quantifies the heterogeneous distribution of SiO_2_-rich aggregates visible in Figure 5, a feature that also accounts for the variations in relative band intensities observed in the surface-sensitive ATR FTIR spectra (Section 2.1). The mean Zn content increased systematically across the series (0.12, 0.15, and 0.18 wt.% for C-Zn1, C-Zn2, and C-Zn3), mirroring the nominal ZnO–SiO_2_ loading trend, although slightly lower than the calculated loadings (0.16, 0.25, and 0.35 wt.% Zn), as expected for sub-wt.% concentrations in a carbon matrix subject to X-ray absorption effects.

### 2.4. Zeta Potential

**Figure 6:**
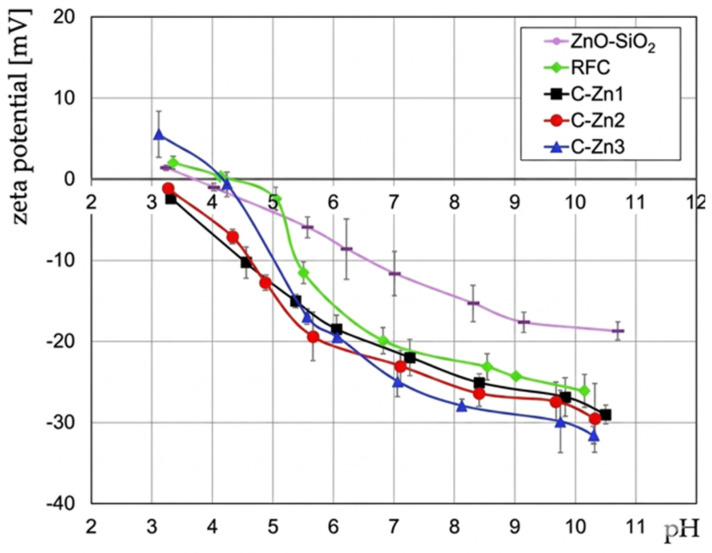
Zeta potential curves versus pH of the studied materials.

The zeta potential (ζ) of the synthesised materials as a function of pH is presented in Figure 6. All samples displayed the expected trend of increasingly negative ζ with rising pH, reflecting the progressive deprotonation of surface silanol (Si–OH), carboxyl (–COOH), and phenolic groups identified by FTIR in Section 2.1 [27,28]. The isoelectric point (IEP) varied markedly among the materials. The pristine RFC exhibited predominantly negative ζ values above its IEP near pH 4, consistent with a surface dominated by strongly acidic carboxyl and phenolic functionalities formed during incomplete carbonisation of the RF network [28]. The ZnO–SiO_2_ modifier exhibited an intermediate IEP near pH 3.5, reflecting the combined influence of two phases with opposite acid–base character: the strongly acidic silica (intrinsic IEP ≈ 1.2–2.5) [29] and the amphoteric ZnO (intrinsic IEP ≈ 9–10) [30]. Positively charged ZnO sites partially neutralise the negative SiO_2_ surface, shifting the composite IEP to a less acidic value.

The C-Zn1 and C-Zn2 composites maintained a negative surface charge across the entire measured pH range (IEP < 3), while C-Zn3, with the highest ZnO–SiO_2_ loading and the most developed mesoporosity, exhibited an IEP near 4.5. The shift in C-Zn3 may reflect a greater contribution of amphoteric Zn-containing surface sites exposed within the more open porous structure, demonstrating that ZnO–SiO_2_ incorporation enables tuneable control of surface charge in this material family. Electrostatic attraction alone, however, cannot fully explain the adsorption trends, since pore accessibility and molecular size effects also play major roles in the adsorption process.

Colloidal stability is governed by |ζ|, with values ≥ ±30 mV generally accepted as the threshold for stable dispersions. The ZnO–SiO_2_ modifier showed the lowest absolute ζ across the entire pH range, indicating weak electrostatic repulsion and a strong tendency toward aggregation. RFC reached approximately −25 mV at pH 8–11, sufficient for moderately stable dispersions. In the neutral-to-alkaline range (pH ≥ 7), C-Zn3 exhibited the largest |ζ|, exceeding ± 30 mV at pH > 9, thus suggesting the highest electrostatic stabilisation of particle dispersions under conditions relevant to water treatment, a behaviour associated with the greater accessible surface area and oxygen-containing surface functionalities.

Overall, the systematic variation of IEP and ζ demonstrates that ZnO–SiO_2_ incorporation enables systematic modulation, dispersion stability, and electrostatic interactions with ionic pollutants, which likely contributes to the observed cationic dye adsorption behaviour discussed in Section 2.5 [31].

### 2.5. Adsorption Studies

**Figure 7:**
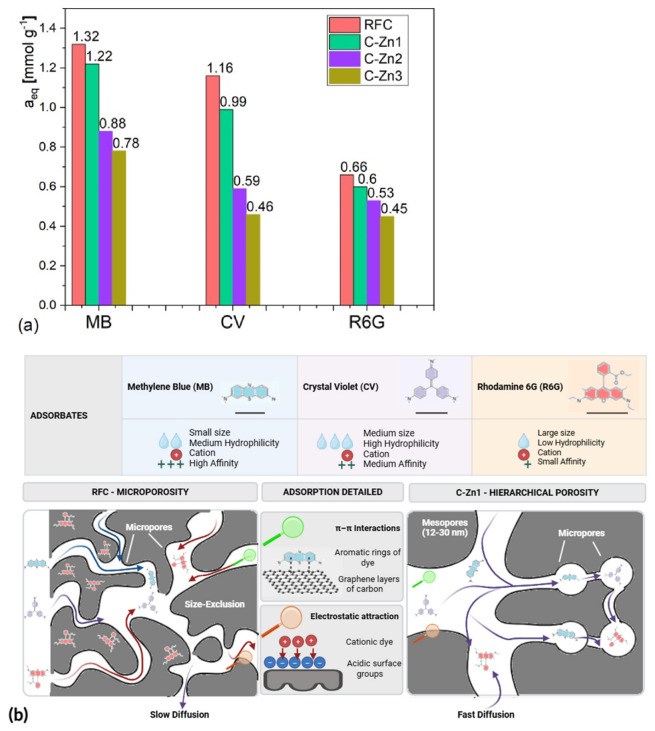
Comparison of adsorption amounts for MB, CV, and R6G (**a**). Comparison of the dye adsorption mechanism on RFC and C-Zn1 (**b**).

Equilibrium and kinetic adsorption of methylene blue (MB), crystal violet (CV), and rhodamine 6G (R6G) on RFC, C-Zn1, C-Zn2, and C-Zn3 was investigated (Figure 7a and Figure S2, Supplementary Materials). The highest uptake for each dye was observed on the unmodified RFC, while the lowest was on C-Zn3, a direct consequence of RFC having the largest specific surface area (545 m^2^ g^−1^ vs. 467–499 m^2^ g^−1^ for the composites, Table 1). Among the modified composites, C-Zn1 displayed sorption capacities very close to RFC despite its smaller surface area (499 m^2^ g^−1^), reflecting the favourable contribution of its hierarchical micro–mesoporosity (dh ≈ 3 nm) for accommodating bulky dye molecules. This indicates that adsorption performance is governed not only by the total surface area, but also by pore accessibility and transport efficiency within the hierarchical pore network. Adsorption likely involves a combination of π–π interactions between the aromatic dye molecules and aromatic carbon domains, together with electrostatic attraction between the cationic dyes and the negatively charged sorbent surface, as confirmed by the zeta potential data (Section 2.4).

Among the three dyes, MB exhibited the highest uptake (0.78–1.32 mmol g^−1^) and R6G the lowest (0.45–0.66 mmol g^−1^). MB is the smallest molecule, facilitating access to micropores. CV is larger and highly soluble in water, factors that may reduce its adsorption affinity relative to MB. R6G is the most hydrophobic (cs = 20 g L^−1^) but also the largest, sterically limiting both capacity and kinetics.

Adsorption isotherms were fitted using the Marczewski–Jaroniec (M–J) equation, also known as the generalised Langmuir (GL) isotherm:

θ = K·ceq^n^/(1 + K·ceq^n^)^m/n^ (1)

where: θ—relative adsorption, θ = a/am, m, n—heterogeneity parameters (0 < m, n ≤ 1), K—the adsorption equilibrium constant connected to the characteristic energy of the energy distribution function. The M–J equation reduces to the generalised Freundlich (GF, n = 1), Langmuir–Freundlich (LF, m = n), Tóth (T, m = 1), and Langmuir (L, m = n = 1) limits [32,33]. The fitted parameters (Table S5) showed a heterogeneity effect for all systems, with MB adsorption best described by the generalised Freundlich limit on all sorbents and the remaining dye/sorbent combinations by the Tóth limit. Fit quality was excellent (R^2^ = 0.948–0.999, SD = 0.008–0.065), and the estimated capacities closely matched the experimental values. Lower m and n parameters for the composites relative to RFC confirm a broader adsorption-energy distribution arising from the coexistence of micro- and mesoporous domains together with Zn-containing surface regions.

The equilibrium constants (log K) quantify the affinity of each dye–sorbent pair. For MB, RFC and C-Zn1 shared a high log K = 0.98, consistent with strong π–π and electrostatic interactions. The anomalously low apparent K value for C-Zn2 likely reflects the strong heterogeneity of the fitted adsorption-energy distribution associated with the low m parameter. For CV, RFC again showed the strongest affinity (log K = 4.09), with progressively lower values for C-Zn1–C-Zn3 (2.11–1.69) as the inorganic content increased. For R6G, log K values were moderate and similar across materials (1.32–1.57), indicating that adsorption is dominated by the bulky molecular geometry rather than by surface chemistry. Among the composites, C-Zn1 consistently combined high capacities with strong K values, demonstrating that the lowest ZnO–SiO_2_ loading yields a hierarchical pore structure most effective for all three cationic dyes.

Kinetic studies confirmed the order MB > CV > R6G in adsorption rate (Figure S4), and identified C-Zn1 as the fastest sorbent and RFC as the slowest, reflecting the diffusional limitation imposed by the purely microporous RFC framework. Bangham analysis (Figure S3) yielded slopes of 0.84–0.99 for all dye/sorbent pairs [34], suggesting that intraparticle diffusion is involved but is not the sole rate-limiting step. Among the simple kinetic models (FOE, SOE, MOE, f-MOE, m-exp; Tables S6 and S7), the multi-exponential (m-exp) equation provided the best fit (SD(c)/c_0_ = 0.106–0.733%; 1 − R^2^ = 1.2 × 10^−5^ − 7.9 × 10^−4^).

The m-exp results indicate that adsorption proceeds in three stages for MB and two stages for CV and R6G, dictated by the porous and heterogeneous nature of the sorbents. Stage 1 corresponds to rapid surface binding, stage 2 to diffusion into micropores, and stage 3 (MB only) to slow filling of the most energetic sites. C-Zn1 yielded the highest rate constants (expressed as logk) for MB of log k_1_ = −1.816, log k_2_ = −2.295, log k_3_ = −3.046 versus log k_1_ = −1.957, log k_2_ = −2.529, log k_3_ = −3.311 for RFC, and the shortest half-times for all dyes (t½ = 102–145 min for C-Zn1 vs. 149–504 min for RFC). The mesopore network created by the ZnO–SiO_2_ modifier therefore substantially accelerates diffusional transport without proportionally sacrificing equilibrium capacity. Figure 7b illustrates the resulting mechanistic contrast between RFC (microporous, slow diffusion) and C-Zn1 (hierarchical, fast transport).

All adsorption experiments were conducted at the natural pH of the dye solutions (~6.5–7.0 for MB and CV, and ~5.5 for R6G), without external pH adjustment. The zeta potential data (Section 2.4) confirmed that all composites maintained a strongly negative surface charge across pH 3–9, with IEPs below 4.5 for C-Zn1 and C-Zn2. Since MB and CV carry permanent positive charges, electrostatic attraction toward the sorbent surface is expected to operate across the investigated near-neutral pH range. A systematic pH-dependent study will form part of a follow-up investigation focused on application conditions.

The MB capacity of C-Zn1 (1.22 mmol g^−1^ = 390 mg g^−1^) is competitive with or superior to most RF-derived carbons and ZnO/carbon hybrids reported in the literature (Table 2), while the non-leaching character of the present composites (Section 2.6) constitutes a significant additional advantage not shared by many reported ZnO–carbon systems.

**Table 2:** Comparison of methylene blue (MB) adsorption capacities: carbon-based and ZnO-containing adsorbents.

Material	Synthesis Approach (S_BET_ [m^2^ g^−1^])	q_max_ MB [mg g^−1^]/[mmolg^−1^]	Isotherm/Kinetic Model	Zn Leaching Assessed	Reference
RFC (unmodified RF carbon)	RF gelation + carbonisation 800 °C, no template (545)	423/1.32	Generalised Freundlich (M–J)	—	This work
ZnO–SiO_2_/C (C-Zn1, best composite)	RF gelation templated with ZnO–SiO_2_/ORISIL-380, carbonisation 800 °C (499)	390/1.22	Generalised Freundlich (M–J)	Not detected (agar well diffusion, 4 bacterial strains)	This work
AC–ZnO (biomass-based activated carbon)	Biomass-derived AC + ZnO NPs in situ via zinc acetate incorporation (n.r.)	138–150/0.43–0.47	Dubinin–Radushkevich	Not assessed	[7]
ZnO/biochar (peanut shell, ZnCl_2_ activation)	One-step pyrolysis, ZnCl_2_ as activating agent and ZnO precursor, O-limited conditions (832)	826/2.58	Langmuir	Not assessed	[35]
ZnO–biochar (ball milling)	Ball milling of ZnO NPs with biochar. No high- temp. synthesis (n.r.)	~150/~0.47	Langmuir	Not assessed	[36]
Formaldehyde resin AC (NaOH activation)	Formaldehyde resin pyrolysis + NaOH chemical activation (626)	152/0.47	Langmuir	—	[37]
RF carbon gel (CO_2_-activated)	RF polycondensation, ambient-pressure drying, CO_2_ activation (514–745)	n.r./n.r.	PSO kinetics. Thomas model (dynamic)	—	[38]
ZnO-RGO nanocomposite	ZnO NPs + reduced graphene oxide, precipitation method (2–7)	88–105/0.27–0.33	Langmuir	Not assessed	[39]
ZnCl_2_-AC (shaddock peel)	Shaddock peel + ZnCl_2_ activation, carbonisation 1000 °C (2399)	870/2.71	Langmuir	—	[40]

### 2.6. Evaluation of Zinc Leaching Character by Agar Well Diffusion Assay

**Figure 8:**
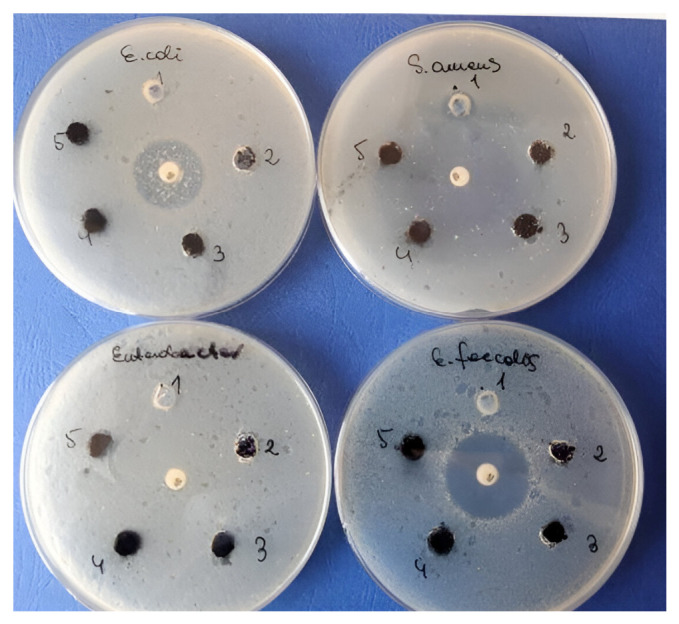
Bacterial growth inhibition of *S. aureus* ATCC 25923, *E. faecalis* ATCC 29212, *E. coli* ATCC 25922, and *E. cloacae* NCTC 13406 by ZnO–SiO_2_ (1), RFC (2), C-Zn1 (3), C-Zn2 (4), and C-Zn3 (5) as well as amoxicillin (centre).

The agar well diffusion method was used to assess whether the synthesised materials release biologically active antibacterial species into the surrounding medium. The assay was performed against two Gram-positive bacterial strains: *Staphylococcus aureus* ATCC 25923, *Enterococcus faecalis* ATCC 29212, and two Gram-negative strains: *Escherichia coli* ATCC 25922, *Enterobacter cloacae* NCTC 13406. The results are presented in Figure 8.

No bacterial growth inhibition zones were observed for any of the carbon-containing materials (RFC, C-Zn1, C-Zn2, and C-Zn3) against any tested bacterial strain. In contrast, the ZnO–SiO_2_ modifier alone produced a narrow inhibition zone of 9 mm against *S. aureus*. Amoxicillin discs (10 µg) yielded inhibition zones of 20–32 mm for *S. aureus*, *E. faecalis*, and *E. coli*, confirming the validity and sensitivity of the assay. *E. cloacae* exhibited resistance to amoxicillin under the applied experimental conditions.

The absence of inhibition zones for the carbon composites indicates that no biologically active concentration of diffusible antimicrobial species was released under the conditions of the agar diffusion assay. This behaviour is consistent with the structural characterisation results: FTIR analysis suggests strong interaction between Zn-containing species and the silica framework (Section 2.1), while SEM observations indicate partial embedding of the inorganic phase within the carbon matrix (Section 2.3). In combination with the relatively low ZnO loading of the composites (0.20–0.42 wt.%, Table 2), these factors may contribute to limiting the release of soluble zinc species into the surrounding medium.

No visible bacterial overgrowth was observed directly on the surface of the carbon-containing materials during the assay. Although the agar diffusion method does not permit a quantitative evaluation of microbial adhesion or biofilm formation, the absence of visible surface colonisation under the applied conditions is favourable from the perspective of potential water-treatment applications.

It should also be noted that the agar well diffusion assay specifically detects soluble antimicrobial species and does not assess the physical retention of bacterial cells by the porous sorbent structure. The high specific surface area (467–545 m^2^ g^−1^), hierarchical micro–mesoporous architecture (Section 2.2), and surface functionality of the composites may potentially contribute to bacterial retention through surface and pore-structure interactions, a mechanism distinct from antibacterial chemical activity and not addressed in the present study. Further investigation of bacterial removal under dynamic filtration conditions will therefore be required.

From an application standpoint, the non-leaching character of the composites is a critical positive attribute: uncontrolled Zn^2+^ release raises environmental and regulatory concerns in drinking water treatment, which suggests that any potential Zn release remains below the threshold required for detectable antibacterial activity. This demonstrates that these composites do not introduce secondary metal contamination into the treated water.

## 3. Materials and Methods

### 3.1. Materials

Hydrophilic fumed silica ORISIL 380 (S_BET_ = 378 m^2^g^−1^, DK Orisil Plant, Ltd., Kalush, Ukraine), zinc acetate dihydrate, Zn(CH_3_COO)_2_·2H_2_O (ACS reagent, ≥98%, Sigma-Aldrich), resorcinol (99.9%, Chimlaborreativ, Brovary, Ukraine), and a 37% aqueous solution of formaldehyde (stabilised with about 10% methanol, Sigma-Aldrich, Darmstadt, Germany) were used in the synthesis of the composites. Double-distilled water was used as the solvent. Crystal violet (CV; POCH Gliwice, Gliwice, Poland), rhodamine 6G (R6G, Sigma, Steinheim, Germany), and methylene blue (MB, Sigma-Aldrich, Darmstadt, Germany) were used to test the effectiveness of dye removal from aqueous solutions.

### 3.2. Synthesis of Composites

The modification of fumed silica with zinc acetate was performed using a liquid-phase method assisted by ultrasonic treatment for 30 min. The component ratio was maintained at 0.2 mmol of zinc acetate per gram of SiO_2_. Subsequent heat treatment in air at 600 °C (1 h) resulted in the oxidative decomposition of zinc acetate, leading to the formation of zinc oxide nanostructures on the silica surface. The resulting material is referred to as the ZnO–SiO_2_ sample.

An aqueous dispersion of the modified silica (5 wt.%) was prepared using an ultrasonic disperser. Resorcinol and formaldehyde were mixed in a molar ratio of 1:2, after which the silica suspension was added to the solution and stirred magnetically for 30 min until complete dissolution of the reagents. The synthesis was performed without the addition of an external alkaline catalyst (e.g., Na_2_CO_3_). The pH of the mixtures was approximately 4.5–5.5. The absence of a strong base allowed for a slower, more controlled polymerisation process. The resulting homogeneous mixture was transferred to a closed plastic container and kept in a thermostat at 85 °C for 20 h to promote polymerisation. The obtained gels were subsequently dried in air at 85 °C for 24 h for the evaporation of the solvent (water) and residual formaldehyde, resulting in a consistent xerogel structure. The dried samples were mechanically ground and carbonised in a vertical quartz reactor under a nitrogen flow of 100 mL min^−1^. The heating rate was 10 °C min^−1^ up to 800 °C, with an isothermal hold for 2 h at the final temperature, followed by cooling under nitrogen. The carbonised composites were labelled as C-Zn1, C-Zn2, and C-Zn3.

The control sample, denoted as RFC, was synthesised under identical conditions but without the addition of the Zn-modified silica dispersion. No additional catalyst was used. For the control RFC sample, the carbon yield was 52% relative to the dry xerogel mass. For the composite samples, the total residual mass after carbonization was 55% for C-Zn1, 61% for C-Zn2, and 68% for C-Zn3. The initial and calculated final compositions of all samples are summarised in Table 3.

The ZnO and SiO_2_ contents in the final composites (Table 3) were calculated from the nominal inorganic mass added to each reaction mixture and the gravimetric carbonisation yield, assuming complete retention of the inorganic ZnO–SiO_2_ phase during pyrolysis under a nitrogen atmosphere, consistent with the thermally stable Si–O–Zn bonding confirmed by FTIR.

### 3.3. Instruments and Measurements

The powder X-ray diffraction (XRD) patterns were recorded with CuKα radiation (λ = 1.5418 Å) using an Empyrean diffractometer (Malvern PANalytical, Malvern, UK, 2012) in the reflection-transmission stage and reflection geometry. The spectra were acquired over a 2θ range of 2–80° with a step size of 0.01°.

The ATR FTIR spectra of the synthesised carbon materials were registered in the range of 400–4000 cm^−1^ using a Tensor 27 apparatus (Bruker, Ettlingen, Germany). For the analysis, the samples were finely ground with anhydrous KBr powder (mass ratio of 1:200 for ZnO–SiO_2_ and 1:450 for carbon composites) and subsequently pressed into thin, homogenous pellets.

The surface morphology of the samples was studied by field emission scanning electron microscopy (SEM, Quanta™ 3D FEG, FEI, Hillsboro, OR, USA) operating at a voltage of 30.0 kV.

Low-temperature (77.4 K) N_2_ adsorption–desorption isotherms were measured on a Micromeritics ASAP 2420 analyser after outgassing at 200 °C for 12 h. The apparent BET surface area S_BET_ was obtained from the Brunauer–Emmett–Teller equation, and the total pore volume Vp from the single-point uptake at p/p_0_ ≈ 0.98 (density-conversion factor 0.0015468 cm^3^(liq) cm^−3^(STP)).

The micropore volume and non-microporous surface area were determined by the comparison-plot (αs) method, which suits microporous carbons with a graphite-like surface and avoids any regularised inversion. The isotherm is expressed as Γ(p) = Γ_micro_ + A· α_s_(p), where α_s_(p) is the reduced adsorption on a nonporous graphitised-carbon reference normalised at p/p_0_ = 0.4 [26]. The plot of adsorbed amount versus αs was fitted over p/p_0_ = 0.07–0.5, above micropore filling and below capillary condensation, giving V_micro_ from the intercept (V_micro_ = Γ_micro_ × 0.0015468) and S_ext_ from the slope (S_ext_ = 2.057 × slope), with S_micro_ = S_BET_ − S_ext_.

These micropore volumes were confirmed by the Harkins–Jura t-plot and an SCV/SCR analysis (slit/cylindrical/void kernel), all three agreeing to within 12%. The SCV/SCR mesopore/macropore split is given in the Supplementary Materials, with the caveat that N_2_ adsorption at 77 K resolves pores only up to R ≈ 50 nm.

Zeta potential analysis was carried out using a Zetasizer NanoZS (Malvern Instruments, Malvern, UK,). Sample preparation began with the preparation of a suspension in a 500 cm^3^ beaker, into which a 10^−2^ mol/dm^3^ NaCl background electrolyte and 0.05 g of the pre-weighed solid sample were introduced. The resulting suspension was sonicated for 2 min using a Sonics Vibra Cell ultrasonicator (Sonics & Materials, Inc., Newtown, CT, USA). Following ultrasonication, the suspension was divided into seven portions, and the pH of each portion was adjusted to values between 3 and 9 using a pH meter (Metrohm, Herisau, Switzerland). Zeta potential values were determined from electrophoretic mobility data calculated by the instrument software using the Smoluchowski equation. Each measurement was performed in six replicates, and the reported values represent the corresponding averages.

### 3.4. Adsorption Studies

Sorption tests were conducted for the cationic dyes methylene blue (MB), crystal violet (CV), and rhodamine 6G (R6G), with their basic properties summarised in Table S1 (Supplementary Materials). Adsorption equilibrium studies were carried out in Erlenmeyer flasks. The volume of the adsorbate solutions was 50 cm^3^, and the mass of adsorbents was 50 mg. Prepared samples were shaken in an incubator shaker for 4 days (25 °C, 110 rpm) to achieve adsorption equilibrium. Subsequently, the absorbance of the solutions (after adsorption) was measured using a Cary 4000 UV–Vis spectrophotometer (Varian, Belrose, Australia). The peak maxima were observed at wavelengths of 664 nm, 582 nm, and 526 nm for MB, CV, and R6G, respectively.

The adsorption isotherms were fitted using the Marczewski–Jaroniec (M–J) isotherm equation, also known as the generalised Langmuir (GL) isotherm equation (see Equation 1 in Section 2.5). Adsorption rate studies were performed using a UV–Vis spectrometer Cary 100 (Varian, Melbourne, Victoria, Australia) equipped with a flow cell. The samples were placed in a thermostatic vessel connected to a mechanical stirrer (EUROSTAR 20, IKA, Poznan, Poland) (110 rpm) and a thermostat (Ecoline RE207, Lauda, Lauda-Königshofen, Germany) (25 °C), after which the adsorbate solutions were added. The initial concentrations of the dyes were 0.024 mmol/L. Adsorption rate data were then analysed using several adsorption kinetics equations, which are listed in Table S2 (Supplementary Materials).

### 3.5. Antibacterial Activity Determination

Antimicrobial properties of the tested materials (ZnO–SiO_2_, RFC, C-Zn1, C-Zn2, C-Zn3) were determined by the agar well diffusion method. Four strains of bacteria, Gram-positive *Staphylococcus aureus* ATCC 25923, *Enterococcus faecalis* ATCC 29212, and Gram-negative *Escherichia coli* ATCC 25922, *Enterobacter cloacae* NCTC 13406, were used. Bacterial cultures were diluted in liquid Mueller Hinton (MH) medium to a density of 0.5 McFarland (1.5 × 10^8^ CFU/mL), added to soft agar, and poured onto the surface of an MH agar plate. After the agar solidified, wells with a diameter of 6 mm were made using a sterile cork borer. Then, 10 mg of each tested material (RFC, C-Zn1, C-Zn2, C-Zn3, and ZnO–SiO_2_) was suspended in 50 µL of sterile distilled water and introduced into each well. For comparison, discs with amoxicillin (10 µg, BioMaxima S.A., Lublin, Poland) were used. Plates were incubated for 18 h at 37 °C.

## 4. Conclusions

A series of hierarchical ZnO–SiO_2_/carbon composites was synthesised by the carbonisation of resorcinol–formaldehyde gels containing chemically anchored ZnO–SiO_2_ aggregates, and their structural, textural, electrokinetic, and adsorption properties were systematically characterised. The principal novelty is the dual role of the inorganic modifier: calcination of zinc acetate on fumed silica at 600 °C yields X-ray-amorphous ZnO integrated into the silica network via Si–O–Zn bonds, which simultaneously acts as an in situ porogen during carbonisation and provides a chemically stable Zn-containing surface phase.

Increasing the ZnO–SiO_2_ loading progressively developed a substantial non-microporous pore volume (non-microporous fraction 0.54–0.63, versus 0.09 for the unmodified RFC) while preserving the micropore network (V_micro_ = 0.156–0.172 cm^3^ g^−1^, S_micro_ = 410–452 m^2^ g^−1^). The best-performing composite, C-Zn1, achieved adsorption capacities of 1.22, 1.04, and 0.63 mmol g^−1^ for methylene blue, crystal violet, and rhodamine 6G—close to the unmodified RFC—while adsorbing up to 3.5-fold faster, demonstrating that the additional mesoporosity shortens diffusion paths to the high-capacity micropores without sacrificing equilibrium uptake.

Crucially, agar well-diffusion tests did not reveal the release of biologically active concentrations of zinc species from any composite, confirming that Si–O–Zn anchoring and encapsulation within the carbon matrix effectively immobilises the metal phase. The combination of high adsorption capacity, accelerated kinetics, a permanently negative surface charge across pH 3–9, and confirmed non-leaching character under aqueous conditions constitutes a well-defined set of properties for application in water purification, and distinguishes the present materials from ZnO/carbon composites prepared by simple physical mixing or impregnation.

Further work will focus on extending the ZnO loading range to activate photocatalytic functionality and on evaluating regeneration performance under multi-cycle conditions.

## Figures and Tables

**Table 1:** Textural characteristics of the composites.

Sample	S_BET_ [m^2^g^−1^]	S_micro_ [m^2^g^−1^]	S_ext_ [m^2^g^−1^]	V_p_ [cm^3^g^−1^]	V_micro_ [cm^3^g^−1^]	V_p_ − V_micro_ [cm^3^g^−1^]	V_micro_/V_p_	D_p_ [nm]
C-Zn1	499	452	46	0.373	0.172	0.201	0.46	2.99
C-Zn2	467	410	57	0.416	0.156	0.260	0.37	3.56
C-Zn3	473	418	56	0.425	0.156	0.269	0.37	3.59
RFC	545	523	22	0.225	0.204	0.021	0.91	1.65

**Table 3:** Initial composition and calculated final composition of the samples.

Sample	Resorcinol [g]	Formalin [g]	SiO_2_ Suspension [g]	H_2_O [g]	SiO_2_(f) [wt.%]	ZnO(f) [wt.%]	C(f) [wt.%]
C-Zn1	10.00	15.04	20.00	~27.1	12.39	0.20	87.4
C-Zn2	10.00	15.04	35.00	~41.2	19.93	0.32	79.7
C-Zn3	10.00	15.03	50.00	~55.5	26.20	0.42	73.4
RFC	10.00	15.04	–	~35.0	–	–	100

## Data Availability

The original contributions presented in this study are included in the article/Supplementary Material. Further inquiries can be directed to the corresponding author(s).

## Supplementary Materials

The following supporting information can be downloaded at: https://www.mdpi.com/article/10.3390/molecules31122079/s1, Table S1. Basic properties of methylene blue, crystal violet, and rhodamine 6G; Table S2. Adsorption kinetics equations used; Table S3. The t-plot fit parameters (Harkins–Jura); Table S4. Validation of Vmicro across three independent methods; Table S5. Marczewski–Jaroniec isotherm parameters; Table S6. Relative standard deviations for kinetic models; Table S7. Multi-exponential equation parameters; Figure S1. The t-plot curves; Figure S2. Adsorption isotherms; Figures S3–S5. Adsorption kinetics and Bangham plots. References [41,42,43,44,45,46,47] are cited in the Supplementary Materials.

## References

[1] Quintas Salamba M., Melo R.L.F., da Silva Aires F.I., de Matos Filho J.R., Nascimento Dari D., Luz Lima F.L., Ferreira Alcântara Araújo S., da Costa Silva L., Fernandes da Silva L., Alexandre Chirindza E. (2025). Porosity of Activated Carbon in Water Remediation: A Bibliometric Review and Overview of Research Perspectives. ACS EST Water.

[2] Thuy Trang Truong T., Tra My Tran T., Kim Chi Tran T., Ngan Vu T., Diu Dinh T., Tran L., Minh Viet Nguyen T., Thao Truong T., Hai Yen Doan T., Duc Pham T. (2023). Highly Adsorptive Removal of Molecular Diclofenac and Bacteria Using Polycation Modified ZnO/SiO2 Nanocomposites. J. Mol. Liq..

[3] Mian M.M., Zhu J., Jiang X., Deng S. (2025). Recent Advances in Activated Carbon Driven PFAS Removal: Structure-Adsorption Relationship and New Adsorption Mechanisms. Front. Environ. Sci. Eng..

[4] Li J., Holze R., Moyo S., Wang S., Li S., Tang T., Chen X. (2021). Three-Dimensional Hierarchical Porous Carbon Derived from Natural Resources for Highly Efficient Treatment of Polluted Water. Environ. Sci. Eur..

[5] Zieliński B., Miądlicki P., Przepiórski J. (2022). Development of Activated Carbon for Removal of Pesticides from Water: Case Study. Sci. Rep..

[6] Thommes M., Kaneko K., Neimark A.V., Olivier J.P., Rodriguez-Reinoso F., Rouquerol J., Sing K.S.W. (2015). Physisorption of Gases, with Special Reference to the Evaluation of Surface Area and Pore Size Distribution (IUPAC Technical Report). Pure Appl. Chem..

[7] Salah W., Djeridi W., Houas A., Elsellami L. (2024). Synergy between Activated Carbon and ZnO: A Powerful Combination for Selective Adsorption and Photocatalytic Degradation. Mater. Adv..

[8] Cruz G.J.F., Gómez M.M., Solis J.L., Rimaycuna J., Solis R.L., Cruz J.F., Rathnayake B., Keiski R.L. (2018). Composites of ZnO Nanoparticles and Biomass Based Activated Carbon: Adsorption, Photocatalytic and Antibacterial Capacities. Water Sci. Technol..

[9] Liu Z., Yu X., Zhou Z., Zhou J., Shuai X., Lin Z., Chen H. (2023). 3D ZnO/Activated Carbon Alginate Beads for the Removal of Antibiotic-Resistant Bacteria and Antibiotic Resistance Genes. Polymers.

[10] Alanazi A.G., Habila M.A., ALOthman Z.A., Badjah-Hadj-Ahmed A.-Y. (2024). Synthesis and Characterization of Zinc Oxide Nanoparticle Anchored Carbon as Hybrid Adsorbent Materials for Effective Heavy Metals Uptake from Wastewater. Crystals.

[11] Espitia P.J.P., Soares N.D.F.F., Coimbra J.S.D.R., De Andrade N.J., Cruz R.S., Medeiros E.A.A. (2012). Zinc Oxide Nanoparticles: Synthesis, Antimicrobial Activity and Food Packaging Applications. Food Bioprocess Technol..

[12] U.S. Food and Drug Administration 21 CFR Part 182—Substances Generally Recognized as Safe. eCFR, Updated 28 May 2026. https://www.ecfr.gov.

[13] Xin Z., He Q., Wang S., Han X., Fu Z., Xu X., Zhao X. (2022). Recent Progress in ZnO-Based Nanostructures for Photocatalytic Antimicrobial in Water Treatment: A Review. Appl. Sci..

[14] Wei W., Tang Z., Zhou Z., Zuo L., Wang Z., Li L., Yang Y. (2025). ZnO-Doped Lignin-Based Carbon as a Catalyst for Ciprofloxacin Photocatalytic Degradation. Int. J. Biol. Macromol..

[15] Zhou D.-Y., Pan G.-Y., Xu M.-L., He X., Li T., Liu F.-T., Jiang F.-H., Li K. (2023). Metal–Organic Framework-Derived Porous Carbon-Mediated ZnO–Nano-ZnO Core–Shell Structure with Excellent Photocatalytic Activity. CrystEngComm.

[16] Nasrollahzadeh M.S., Hadavifar M., Ghasemi S.S., Arab Chamjangali M. (2018). Synthesis of ZnO Nanostructure Using Activated Carbon for Photocatalytic Degradation of Methyl Orange from Aqueous Solutions. Appl. Water Sci..

[17] Pham T.-D., Truong T.-T.-T., Nguyen H.-L., Hoang L.-B.-L., Bui V.-P., Tran T.-T.-M., Dinh T.-D., Le T.-D. (2022). Synthesis and Characterization of Novel Core–Shell ZnO@SiO_2_ Nanoparticles and Application in Antibiotic and Bacteria Removal. ACS Omega.

[18] Yang C., Wang J., Fan H., Hu Y., Shen J., Shangguan J., Wang B. (2018). Activated Carbon-Assisted Fabrication of Cost-Efficient ZnO/SiO_2_ Desulfurizer with Characteristic of High Loadings and High Dispersion. Energy Fuels.

[19] Gun’ko V.M., Bogatyrov V.M., Oranska O.I., Borysenko L.I., Skubiszewska-Zięba J., Książek A., Leboda R. (2013). Structural Features of ZnxOy/Nanosilica Composites. Appl. Surf. Sci..

[20] Nazarkovsky M., Czech B., Żmudka A., Bogatyrov V.M., Artiushenko O., Zaitsev V., Saint-Pierre T.D., Rocha R.C., Kai J., Xing Y. (2021). Structural, Optical and Catalytic Properties of ZnO-SiO2 Colored Powders with the Visible Light-Driven Activity. J. Photochem. Photobiol. A Chem..

[21] Galaburda M., Nazarkovsky M., Osipiuk K., Czech B., Borysenko M.V., Gładysz-Płaska A., Lipke A., Marinkovic B.A., Navarro R.C.S., Deryło-Marczewska A. (2024). Enhanced Photocatalytic Degradation of Antiviral Drugs Lopinavir and Ritonavir by Ni Doped ZnO/SiO2 Nanocomposites. J. Environ. Chem. Eng..

[22] Nazarkovsky M.A., Bogatyrov V.M., Czech B., Galaburda M.V., Wójcik G., Kolomys O.F., Strelchuk V.V., Malysheva M.L., Oranska O.I., Gun’ko V.M. (2017). Synthesis and Properties of Zinc Oxide Photocatalyst by High-Temperature Processing of Resorcinol-Formaldehyde/Zinc Acetate Mixture. J. Photochem. Photobiol. A Chem..

[23] Galaburda M., Sternik D., Chrzanowska A., Oranska O., Kovalov Y., Derylo-Marczewska A. (2024). Physicochemical and Adsorption Characterization of Char Derived from Resorcinol–Formaldehyde Resin Modified with Metal Oxide/Silica Nanocomposites. Materials.

[24] Boehm H.P. (2002). Surface oxides on carbon and their analysis: A critical assessment. Carbon.

[25] Goncharuk O., Bogatyrov V., Kazakova O., Galaburda M., Oranska O., Skwarek E., Waniak-Nowicka H., Janusz W., Gun’ko V. (2019). Silica-Supported Ni_x_O_y_, Zn_x_O_y_ and Mn_x_O_y_ Nanocomposites: Physicochemical Characteristics and Interactions with Water and n-Decane. Bull. Mater. Sci..

[26] Kruk M., Jaroniec M., Sayari A. (1997). Application of Large Pore MCM-41 Molecular Sieves To Improve Pore Size Analysis Using Nitrogen Adsorption Measurements. Langmuir.

[27] Yang R., Wang F., Blunk R.H., Angelopoulos A.P. (2010). Competing Effects of Silanol Surface Concentration and Solvent Dielectric Constant on Electrostatic Layer-by-Layer Assembly of Silica Nanoparticles on Gold. J. Colloid Interface Sci..

[28] Fletcher A.J., Uygur Y., Thomas K.M. (2007). Role of Surface Functional Groups in the Adsorption Kinetics of Water Vapor on Microporous Activated Carbons. J. Phys. Chem. C.

[29] Xu G., Zhang J., Song G. (2003). Effect of Complexation on the Zeta Potential of Silica Powder. Powder Technol..

[30] Degen A., Kosec M. (2000). Effect of pH and Impurities on the Surface Charge of Zinc Oxide in Aqueous Solution. J. Eur. Ceram. Soc..

[31] Yang X., Wan Y., Zheng Y., He F., Yu Z., Huang J., Wang H., Ok Y.S., Jiang Y., Gao B. (2019). Surface Functional Groups of Carbon-Based Adsorbents and Their Roles in the Removal of Heavy Metals from Aqueous Solutions: A Critical Review. Chem. Eng. J..

[32] Marczewski A.W., Jaroniec M. (1983). A New Isotherm Equation for Single-Solute Adsorption from Dilute Solutions on Energetically Heterogeneous Solids. Monatsh. Chem..

[33] Jaroniec M., Marczewski A.W. (1984). Physical Adsorption of Gases on Energetically Heterogeneous Solids I. GeneralizedLangmuir Equation and Its Energy Distribution. Monatsh. Chem..

[34] Aharoni C., Sideman S., Hoffer E. (1979). Adsorption of Phosphate Ions by Collodion-coated Alumina. J. Chem. Technol. Biotechnol..

[35] Nguyen T.L., Nguyen K.V., Dang N.V., Huy T.Q., Linh P.H., Trung N.T., Nguyen V.-T., Thanh D.V. (2023). Facile one-step pyrolysis of ZnO/biochar nanocomposite for highly efficient removal of methylene blue dye from aqueous solution. ACS Omega.

[36] Yu F., Tian F., Zou H., Ye Z., Peng C., Huang J., Zheng Y., Zhang Y., Yang Y., Wei X. (2021). ZnO/biochar nanocomposites via solvent free ball milling for enhanced adsorption and photocatalytic degradation of methylene blue. J. Hazard. Mater..

[37] Yusri A.Z.H., Zaini M.A.A. (2026). Hardened formaldehyde resin-based activated carbons for methylene blue removal. Indian Chem. Eng..

[38] Faizal A.N.M., Halim M.H.A., Zaini M.A.A. (2019). Kinetics and dynamic adsorption of methylene blue by CO_2_-activated resorcinol formaldehyde carbon gels. Carbon Lett..

[39] Hussein S.A., Taha G.M., Adam F.A., Moghazy M.A. (2025). Three different methods for ZnO-RGO nanocomposite synthesis and its adsorption capacity for methylene blue dye removal in a comparative study. BMC Chem..

[40] Singh S., Sahoo S., Sahoo B.C., Dash M., Nayak S., Kar B. (2022). Derivatives of Cinnamic Acid Esters and Terpenic Diversity in Volatiles of Thirty-Six Sand Ginger (*Kaempferia galanga* L.) Accessions of Eastern India Revealing Quality Chemovars. Molecules.

[41] Lagergren S. (1989). Zur Theorie der sogenannten Adsorption gelöster Stoffe. K. Sven. Vetenskapsakad. Handl..

[42] Azizian S. (2004). Kinetic Models of Sorption: A Theoretical Analysis. J. Colloid Interface Sci..

[43] Marczewski A.W. (2007). Kinetics and Equilibrium of Adsorption of Organic Solutes on Mesoporous Carbons. Appl. Surf. Sci..

[44] Marczewski A.W. (2010). Application of Mixed Order Rate Equations to Adsorption of Methylene Blue on Mesoporous Carbons. Appl. Surf. Sci..

[45] Marczewski A.W. (2010). Analysis of Kinetic Langmuir Model. Part I: Integrated Kinetic Langmuir Equation (IKL): A New Complete Analytical Solution of the Langmuir Rate Equation. Langmuir.

[46] Marczewski A.W., Deryło-Marczewska A., Słota A. (2013). Adsorption and Desorption Kinetics of Benzene Derivatives on Mesoporous Carbons. Adsorption.

[47] Haerifar M., Azizian S. (2012). Fractal-Like Adsorption Kinetics at the Solid/Solution Interface. J. Phys. Chem. C.

