# Supplementary material for: Hierarchical Micro–Mesoporous ZnO–SiO2/Carbon Composites: Synthesis, Structural Characterisation, and High-Capacity Adsorption of Cationic Organic Pollutants from Water
Source: Molecules. 2026 Jun 13;31(12):2079. doi: 10.3390/molecules31122079 (PMC13305421; doi:10.3390/molecules31122079)
Supplement: Supplementary file 1 [file molecules-31-02079-s001.zip › molecules-4349856-supplementary.pdf]

molecules

Supplementary Materials

Hierarchical Micro–Mesoporous ZnO–SiO2/Carbon Composites: Synthesis, Structural Characterisation, and High-Capacity Adsorption of Cationic Organic Pollutants from Water

Mariia Galaburda, Małgorzata Wasilewska, Elżbieta Grządka * and Jolanta Kutkowska

* Correspondence: elzbieta.grzadka@mail.umcs.pl

Table S1. Selected physicochemical properties of methylene blue, crystal violet, and rhodamine 6G.

Adsorbate	Structural Formula	Ma [g/mol]	cs b [g/L]	pKa c	m.p. d [°C]	Chemical Safety
Methylene Blue	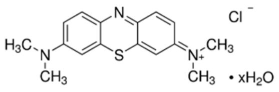	319.85	43.6	>12	100–110	Corrosive; Irritant
Crystal Violet	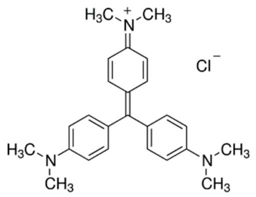	407.98	50	8.64	215	Corrosive; Irritant; Health Hazard; Environmental Hazard
Rhodamine 6G	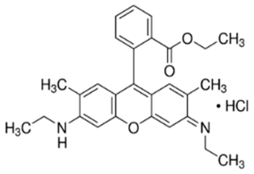	479.01	20	–	263–265	Corrosive; Acute Toxic; Environmental Hazard

Ma—molar mass; cs b—solubility in water at 25 °C; pKa c—ionization constant; m.p. d—melting point.

Table S2. Selected kinetic equations [41-47].

Name and abbreviation	Formula
The First-order equation / pseudo-first-order equation (FOE/PFOE)	$ln(c_{eq} - c) = ln(c_{eq} - c_o) - k_1 t \text{ or } ln(a_{eq} - a) = lna_{eq} - k_1 t$ (2)<br>c - the temporary concentration, a - the actual adsorbed amount, the o and eq subscripts are connected to the initial and equilibrium states, k_1 - the adsorption rate coefficient.
The Second-order equation / pseudo-second-order equation (SOE/PSOE)	$a = a_{eq}[k_2 t/(1 + k_2 t)]$ (3)<br>or $t/a = (1/a_{eq})(1/k_2 + t)$ and $a = a_{eq}[k_2 t/(1 + k_2 t)]$<br>$k_2 = k_{2a} \cdot a_{eq}$ and k_{2a} - the rate coefficients for pseudo-second-order kinetics.

1,2-mixed-order equation (MOE)	$F = a/a_{eq} = \frac{1-exp(-k_1 t)}{1-f_2 exp(-k_1 t)}$ or $ln\left(\frac{1-F}{1-f_2 F}\right) = -k_1 t$ (4)<br>F - the relative adsorption progress in time, $f_2<1$is the normalized share of the second order process in the kinetics. The MOE equation can simplify to the FOE ($f_2=0$) and the SOE ($f_2=1$) type.
Fractal-like MOE equation (f-MOE)	$F = \frac{1 - exp(-k_1 t)^p}{1 - f_2 exp(-k_1 t)^p}$ (5)<br>p - the fractal coefficient.
Multi-exponential equation (m-exp)	$c = \left(c_o - c_{eq}\right)\sum_{i=1}^{n} f_i exp(-k_i t) + c_{eq}$ (6)<br>or $c = c_0 - c_0 u_{eq} \sum_{i=1}^{n} f_i [1 - exp(-k_i t)]$<br>"$i$" - the term of m-exp equation, k_i - the rate coefficient and $u_{eq}=1-c_{eq}/c_o$ - the relative loss of adsorbate from the solution.

As an independent, non-regularised check of the micropore volume, the Harkins–Jura t-plot was constructed for every sample. The statistical film thickness was computed as

$$t = \frac{1}{10}\sqrt{\frac{13.99}{0.034 - lg\left(\frac{p}{p_0}\right)}}$$

and the adsorbed amount was regressed linearly against t in the multilayer region of each isotherm (t = 0.35–0.45 nm for the purely microporous RFC; t = 0.55–0.81 nm for the C-Zn composites). The micropore volume was obtained from the intercept (V_{miCro} = intercept × 0.0015468) and the external surface area from the slope (S_{ext} = slope × 15.47). The V_{miCro} values agree with the SCV/SCR (Table 1) and NLDFT estimates within 10% for all samples (Table S3 and Figure S1), confirming that the reported micropore volumes reflect the genuine micropore structure rather than the choice of numerical inversion scheme.

Table S3. t-Plot fit parameters (Harkins–Jura).

Sample	t-window (nm)	R^2	V_{miro} (cm³/g)	S_{miro} (m²/g)	S_{ext} (m²/g)	S_{BET} (m²/g)
RFC	0.35–0.45	0.9826	0.197	497.0	48.5	545.4
C-Zn1	0.55–0.81	0.9999	0.189	305.0	193.6	498.6
C-Zn2	0.55–0.81	0.9999	0.175	421.8	45.2	467.0
C-Zn3	0.55–0.81	0.9998	0.175	409.8	63.9	473.7

For the hierarchical C-Zn composites, the t-plot S_{ext} values (45–194 m^2 g^{-1}) differ from those obtained by the αs comparison plot (46–57 m^2 g^{-1}, Table 1) because the two methods probe different p/p_0 ranges; the t-plot is used here exclusively as an independent, non-regularised validation of Vmicro, which agrees within 10–12% across all methods (Table S4)

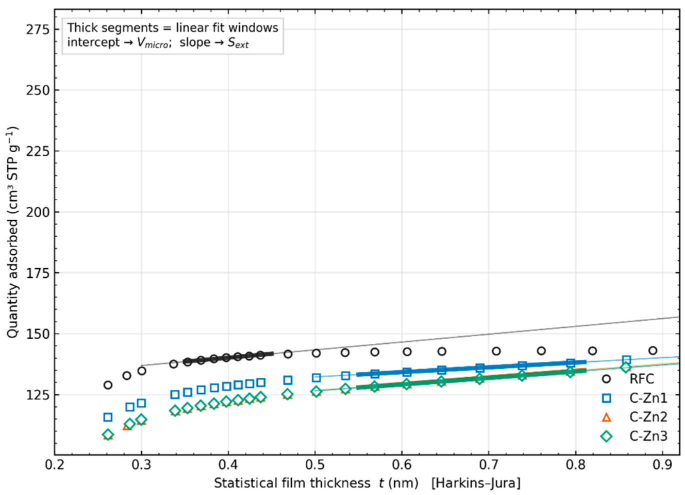

Figure S1. t-Plot curves for RFC and the C-Zn composites.

Table S4. Validation of V_{miCro} across three independent methods.

**Sample**	**V_{miCro} SCV/SCR (this work, cm³/g)**	**V_{miCro} NLDFT (cm³/g)**	**V_{miCro} t-plot (cm³/g)**
RFC	0.217	0.207	0.197
C-Zn1	0.194	0.190	0.189
C-Zn2	0.182	0.185	0.175
C-Zn3	0.181	0.185	0.175

The micropore volume from the primary SCV/SCR analysis (Table 1) is confirmed by two fully independent methods - NLDFT (slit-pore equilibrium kernel) and the non-regularised Harkins–Jura t-plot, with agreement within 10% for all four samples.

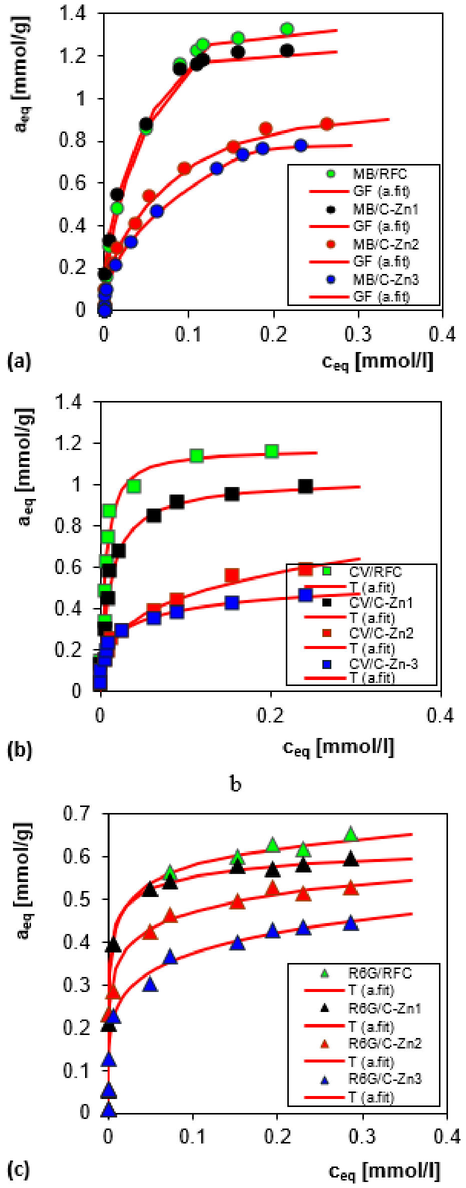

Figure S2. The adsorption isotherms for MB (b), CV (c) and R6G (d) on RFC, C-Zn1, C-Zn2 and C-Zn3 materials.

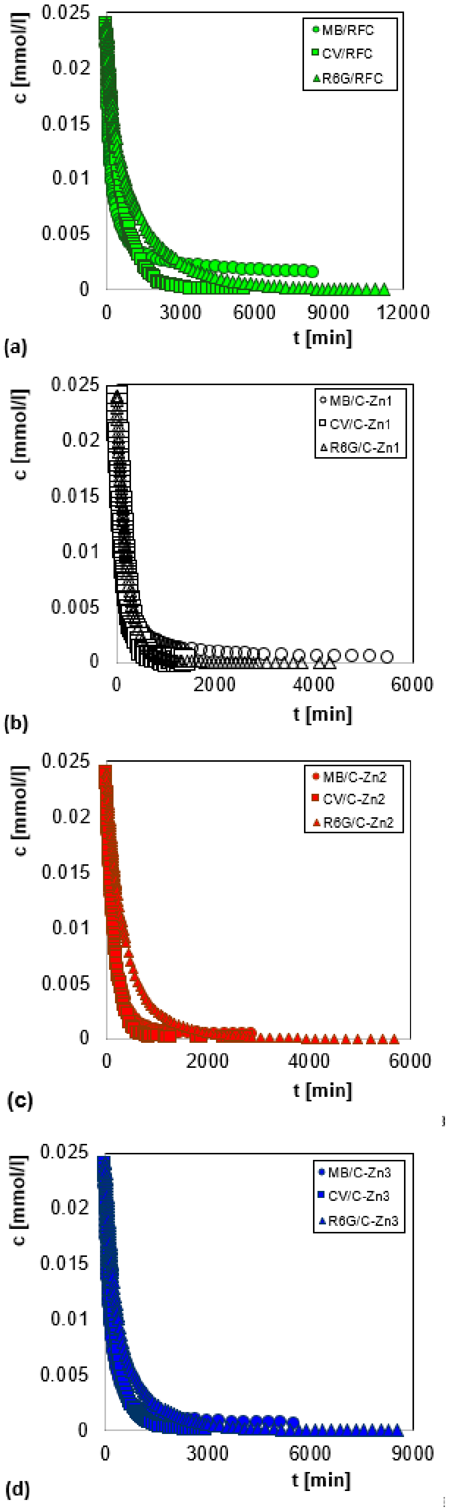

Figure S3. Adsorption kinetics for MB, CV and R6G on RFC (a), C-Zn1 (b), C-Zn2 (c) and C-Zn3 (d) materials showed as changes in concentration over time.

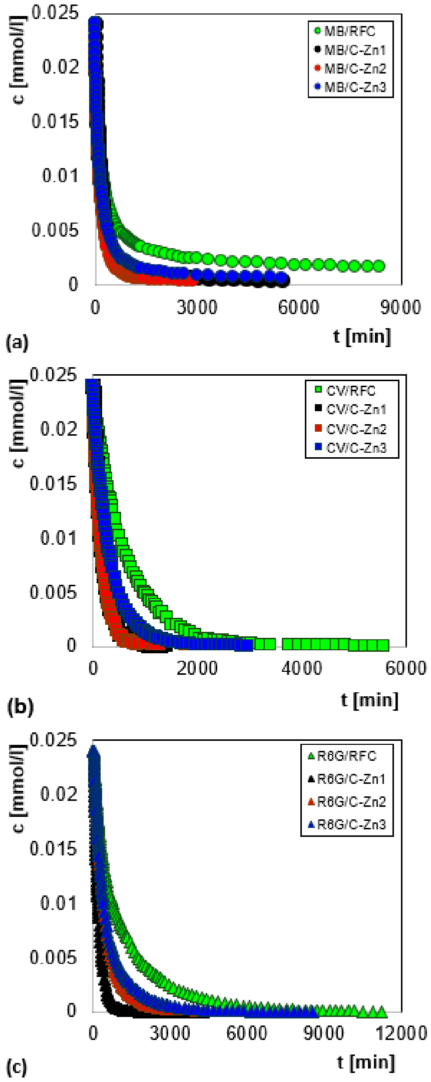

Figure S4. Adsorption kinetics for MB (a), CV (b) and R6G (c) on RFC, C-Zn1, C-Zn2 and C-Zn3 materials showed as changes in concentration over time.

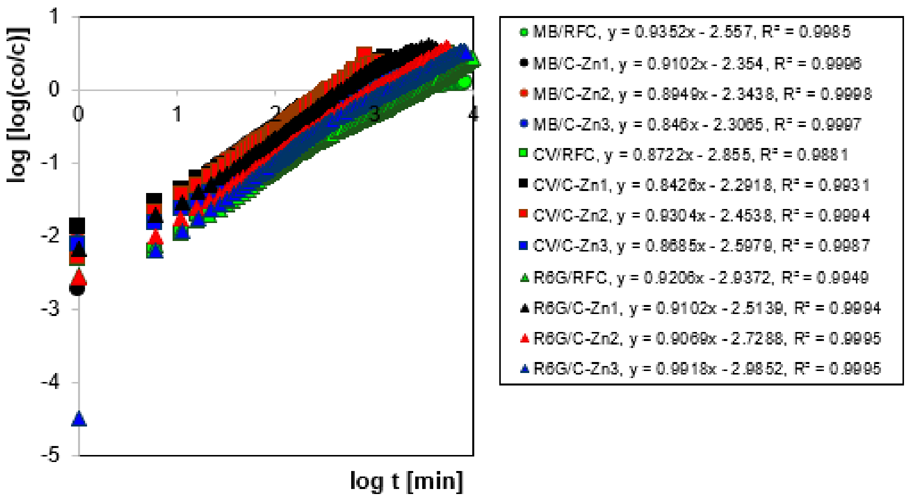

Figure S5. The Bangham plots for adsorption kinetics of MB, CV and R6G on RFC, C-Zn1, C-Zn2 and C-Zn3 materials.

Table S5. Parameters of the M-J eq. for adsorption of MB, CV and R6G on the RFC, C-Zn1, C-Zn2 and C-Zn3 materials.

System	Isotherm Type	a_m	m	n	logK	R^2	SD (a)
MB/RFC	GF	1.32	0.56	1	0.98	0.995	0.040
MB/C-Zn1	GF	1.22	0.44	1	0.98	0.995	0.038
MB/C-Zn2	GF	0.93	0.21	1	-4.67	0.999	0.012
MB/C-Zn3	GF	0.78	0.48	1	0.73	0.999	0.009
CV/RFC	T	1.17	1	0.57	4.09	0.979	0.065
CV/C-Zn1	T	1.04	1	0.20	2.11	0.998	0.017
CV/C-Zn2	T	0.62	1	0.54	1.83	0.995	0.015
CV/C-Zn3	T	0.47	1	0.38	1.69	0.997	0.008
R6G/RFC	T	0.67	1	0.65	1.57	0.974	0.044
R6G/C-Zn1	T	0.63	1	0.86	1.52	0.981	0.036
R6G/C-Zn2	T	0.55	1	0.84	1.45	0.948	0.051
R6G/C-Zn3	T	0.51	1	0.25	1.32	0.979	0.027

Table S6. Relative standard deviations SD(c)/co (%) for chosen equations of adsorption kinetics.

System	m-exp [%]	FOE [%]	SOE [%]	MOE [%]	f-FOE [%]	f-SOE [%]	f-MOE [%]
MB/RFC	0.215	3.329	0.397	0.395	1.588	0.216	0.217
MB/C-Zn1	0.106	2.328	1.887	0.594	1.106	0.526	2.726
MB/C-Zn2	0.169	1.148	4.158	0.271	0.279	1.531	1.540
MB/C-Zn3	0.273	2.152	1.928	0.576	0.968	0.710	0.714
CV/RFC	0.462	0.734	6.125	0.486	0.479	2.367	2.373
CV/C-Zn1	0.733	0.720	6.124	0.717	0.721	2.015	2.047
CV/C-Zn2	0.500	0.523	6.280	0.525	0.522	4.092	2.364
CV/C-Zn3	0.339	0.574	9.380	0.445	0.465	2.633	2.647
R6G/RFC	0.371	3.765	2.826	0.851	0.992	2.463	0.862
R6G/C-Zn1	0.287	0.281	6.241	0.242	0.259	2.114	2.143
R6G/C-Zn2	0.160	1.091	5.317	0.460	0.614	1.637	1.642
R6G/ C-Zn3	0.513	3.931	10.366	0.892	1.767	2.946	2.946

Table S7. Optimized parameters of m-exp eq. for adsorption kinetics of MB, CV and R6G on RFC, C-Zn1, C-Zn2 and C-Zn3 materials.

System	f_1, log k_1	f_2, log k_2	f_3, log k_3	u_{eq}	$t_{1/2}$ [min]	SD(c)/c_o [%]	1-R^2
MB/RFC	0.412; −1.957	0.441; −2.529	0.147; −3.311	0.986	148.99	0.215	5.8×10^{-5}
MB/C-Zn1	0.339; −1.816	0.550; −2.295	0.111; −3.046	1	102.11	0.106	1.2×10^{-5}
MB/C-Zn2	0.086; −1.534	0.806; −2.200	0.108; −2.698	1	107.37	0.169	2.8×10^{-5}
MB/C-Zn3	0.029; 0.146	0.665; −2.101	0.306; −2.742	1	121.21	0.273	2.8×10^{-5}
CV/RFC	0.084; −2.278	0.916; −2.819	-	1	412.12	0.462	7.9×10^{-4}
CV/C-Zn1	0.984, −2.212	0.016, −3.503	-	1	115.31	0.733	5.1×10^{-4}
CV/C-Zn2	0.989, −2.231	0.011, −0.189	-	1	115.95	0.500	2.3×10^{-4}
CV/C-Zn3	0.854; −2.453	0.146; −2.790	-	1	217.28	0.339	5.1×10^{-5}
R6G/RFC	0.642; −3.205	0.358; −2.315	-	1	503.59	0.371	1.1×10^{-4}
R6G/C-Zn1	0.990; −2.320	0.108; −3.428	-	1	144.73	0.287	7.3×10^{-5}
R6G/C-Zn2	0.710; −2.450	0.290; −2.892	-	1	252.16	0.160	2.0×10^{-5}
R6G/C-Zn3	0.604; −2.420	0.396; −3.017	-	1	290.04	0.513	1.0×10^{-4}

References

41. Zur Theorie der sogenannten Adsorption gelöster Stoffe: Lagergreen, S., (Bihang A. K. Svenske Vet. Ak. Handl. 24, II. Nr. 4, S. 49; 1899; Z. physik. Ch. 32, 174–75; 1900.). *Z. Für Chem. Ind. Kolloide* **1907**, *2*, 15–15, doi:10.1007/BF01501332.
42. Azizian, S. Kinetic Models of Sorption: A Theoretical Analysis. *J. Colloid Interface Sci.* **2004**, *276*, 47–52, doi:10.1016/j.jcis.2004.03.048.
43. Marczewski, A.W. Kinetics and Equilibrium of Adsorption of Organic Solutes on Mesoporous Carbons. *Appl. Surf. Sci.* **2007**, *253*, 5818–5826, doi:10.1016/j.apsusc.2006.12.037.
44. Marczewski, A.W. Application of Mixed Order Rate Equations to Adsorption of Methylene Blue on Mesoporous Carbons. *Appl. Surf. Sci.* **2010**, *256*, 5145–5152, doi:10.1016/j.apsusc.2009.12.078.
45. Marczewski, A.W. Analysis of Kinetic Langmuir Model. Part I: Integrated Kinetic Langmuir Equation (IKL): A New Complete Analytical Solution of the Langmuir Rate Equation. *Langmuir* **2010**, *26*, 15229–15238, doi:10.1021/la1010049.
46. Marczewski, A.W.; Deryło-Marczewska, A.; Słota, A. Adsorption and Desorption Kinetics of Benzene Derivatives on Mesoporous Carbons. *Adsorption* **2013**, *19*, 391–406, doi:10.1007/s10450-012-9462-7.
47. Haerifar, M.; Azizian, S. Fractal-Like Adsorption Kinetics at the Solid/Solution Interface. *J. Phys. Chem. C* **2012**, *116*, 13111–13119, doi:10.1021/jp301261h.
